# Acute chest pain - A prospective population based study of contacts to Norwegian emergency medical communication centres

**DOI:** 10.1186/1471-227X-11-9

**Published:** 2011-07-21

**Authors:** Robert Anders Burman, Erik Zakariassen, Steinar Hunskaar

**Affiliations:** 1 National Centre for Emergency Primary Health Care, Uni Health, Kalfarveien 31, 5018 Bergen, Norway; 2 Department of Public Health and Primary Health Care, University of Bergen, Post box 7804, 5020 Bergen, Norway; 3 Department of Research, Norwegian Air Ambulance Foundation, Post box 94, 1441 Drøbak, Norway

**Keywords:** Chest pain, After-hours care, Emergency medical services, Emergencies

## Abstract

**Background:**

Acute chest pain is a frequently occurring symptom in patients with medical emergencies and imposes potentially life threatening situations outside hospitals. Little is known about the epidemiology of patients with acute chest pain in a primary care setting in Norway, and we aimed to obtain more representative data on such patients using data from emergency medical communication centres (EMCCs).

**Methods:**

Data were collected prospectively during three months in 2007 from three EMCCs, covering 816 000 inhabitants. The EMCCs gathered information on every situation that was triaged as a red response (defined as an "acute" response, with the highest priority), according to the Norwegian Index of Medical Emergencies. Records from ambulances and primary care doctors were subsequently collected. International Classification of Primary Care - 2 symptom codes and The National Committee on Aeronautics (NACA) System scores were assigned retrospectively. Only chest pain patients were included in the study.

**Results:**

5 180 patients were involved in red response situations, of which 21% had chest pain. Estimated rate was 5.4 chest pain cases per 1000 inhabitants per year. NACA-scores indicated that 26% of the patients were in a life-threatening medical situation. Median prehospital response time was 13 minutes; an ambulance reached the patient in less than 10 minutes in 30% of the cases. Seventy-six per cent of the patients with chest pain were admitted to a hospital for further investigation, 14% received final treatment at a casualty clinic, while 10% had no further investigation by a doctor ("left at the scene").

**Conclusions:**

The majority of patients with acute chest pain were admitted to a hospital for further investigation, but only a quarter of the patients were assessed prehospitally to have a severe illness. This sheds light on the challenges for the EMCCs in deciding the appropriate level of response in patients with acute chest pain. Overtriage is to some extent both expected and desirable to intercept all patients in need of immediate help, but it is also well known that overtriage is resource demanding. Further research is needed to elucidate the challenges in the diagnosis and management of chest pain outside hospitals.

## Background

Acute chest pain is an important and frequently occurring symptom in patients with medical emergencies outside hospitals [1-3]. Chest pain is often a sign of ischaemic heart disease, although gender, age and comorbidity may modify how acute coronary heart disease presents itself within the individual patient. Acute chest pain may indicate a potentially life threatening situation, but it is also commonly acknowledged that a wide variety of differential diagnosis exists, many with lower health impact and less serious potential [4,5].

In Norway, patients in need of acute medical assistance are recommended to come in contact with the emergency health care system by calling the health specific national three digits emergency number 113, thereby reaching the nearest emergency medical communication centre (EMCC). Similar three digits emergency numbers also exist for the fire department (110) and the police (112). When a call reaches the EMCC, trained nurses use a decision tool, the Norwegian Index of Medical Emergencies [6], to classify the actual medical problem into one of three levels of response, each indicated by a colour code. "Red response" indicates an immediate need of help (potentially or manifest life threatening situation), and will trigger the transmission of a simultaneous radio alarm from the EMCC to both the primary care doctor on-call and the ambulance service in the relevant area.

Little is known about the epidemiology of acute chest pain outside hospitals in Norway. A recent study from a single island municipality documented an incidence of 27 medical emergencies per 1 000 inhabitants per year, with an incidence rate of acute chest pain and suspected myocardial infarction of about 4.8 patients per 1 000 inhabitants per year [7]. Another study examined prehospital diagnosis and treatment of acute myocardial infarction in a single county in Norway [8]. An incidence rate of 5.4 per 1 000 inhabitants per year of acutely ill patients with chest pain or suspected acute myocardial infarction was found.

In a previous study [1] we presented data from three EMCCs after gathering information on every situation that was triaged as a red response, according to the Norwegian Index of Medical Emergencies. The study showed that 90% of the red responses were medical problems with a large variation of symptoms, the remainder being accidents. Severity of illness was classified retrospectively, and showed that 70% of the patients were not in a life-threatening situation.

The aim of the present analyses was to obtain representative data on the epidemiology of acute chest pain outside the hospitals in Norway, by a more detailed investigation of the data from our EMCC study.

## Methods

Three EMCCs, located at Haugesund, Stavanger and Innlandet hospitals, were involved in the study, with the three corresponding districts covering 816 000 inhabitants (18% of the total Norwegian population). Data were collected prospectively from October 1 to December 31 2007.

### Variables

All 19 EMCCs in Norway use a software system called Acute Medical Information System (AMIS) to record all incoming cases. Usage of the AMIS results in an electronic form with registration of each incident (*not *the individual patient). The AMIS form contains information about the incident, the patient (or patients, if more than one patient is involved in the incident) and all available logistics, including date, time of day, and to where the patients are transported ("left at scene", home, casualty clinic, hospital). Prehospital response time is also registered, defined as the time period from when the caller calls 113 until the nearest available ambulance reaches the patient [9,10].

Based on the immediate available information, the EMCC operator (usually a specially trained nurse) gives the incident one clinical criteria code and one response level according to the Index [6]. The Index is based on ideas from the Criteria Based Dispatch system in the US [11], and was first published in 1994. It categorises clinical symptoms, findings and incidents into 39 chapters, and each chapter is subdivided into a red, yellow and green criteria based section, correlating to the appropriate level of response. Red colour is defined as an "acute" response, with the highest priority, and will trigger the transmission of a radio alarm to both the primary care doctor on-call and the ambulance service. Yellow colour is defined as an "urgent" response, with a high, but lower priority, where the patient should be examined as soon as the doctor-on-call is available. Green colour is defined as a "non-urgent" response, with the lowest priority. Chapter 10 in the Index covers the symptom "Chest pain", and usage of the red response section will result in the code A10 - Chest pain (A for "acute"). An example of a criterion leading to a red response will be "chest pain with breathing difficulties", while "pain not particular strong, and the patient feels fine" is defined as a yellow criterion, leading to an urgent response, but with lower priority than red response.

Copies of all AMIS forms involving incidents classified as red response were sent to the project manager every other week throughout the study. The EMCCs also sent copies of ambulance records from all red responses which involved ground or boat ambulances. In cases where doctors on-call, casualty clinics, primary care doctors or air ambulances had been involved, copies of medical records were requested and collected separately. This collection of medical records continued also after the study period, until October 2008. To secure a uniform use of the variables in the AMIS program, a meeting was held between the persons in charge of the participating EMCCs.

The severity of the medical problem was classified using The National Committee on Aeronautics (NACA) Score System based on all available information [12]. In the NACA system, the patient's status is classified from 0 to 7, zero indicating no disease or injury, while seven indicates the patient being dead. NACA score was categorised in the analyses as NACA 0-1 (patient with either no symptoms/injuries or in no need of medical treatment), NACA 2-3 (patient in need of medical help, where value 3 indicates need of hospitalisation, but still not a life-threatening situation), NACA 4-6 (4 is a potentially, and 5 and 6 are definitely, life-threatening medical situations) and NACA 7 (dead person).

Based on information from all available forms and medical records the cases were also classified into symptom groups according to the International Classification of Primary Care - 2 (ICPC - 2) [13]. The analyses presented in the results-section are based on the patients who were given the code A10 - Chest pain. Results on all the clinical categories and symptom groups, are published in a previous article [1].

### Statistical analyses

The statistical analyses were performed using Statistical Package for the Social Sciences (SPSS version 15). Standard univariate statistics, including median and percentiles, were used to characterise the sample. Median, with 25th-75th percentiles, was used to analyse data where normal distribution was not present. Rates are presented as numbers of red responses per 1 000 inhabitants per year with a 95%-confidence interval (CI). Mann-Whitney U test was used for comparing age between males and females, for other comparisons the Pearson Chi-Square test was used. A P-value of < 0.05 was considered statistically significant.

### Ethics and approvals

Approval of the study was given by the Privacy Ombudsman for Research, Regional Committee for Medical Research Ethics, and the Norwegian Directorate of Health.

## Results

**Figure 1:**
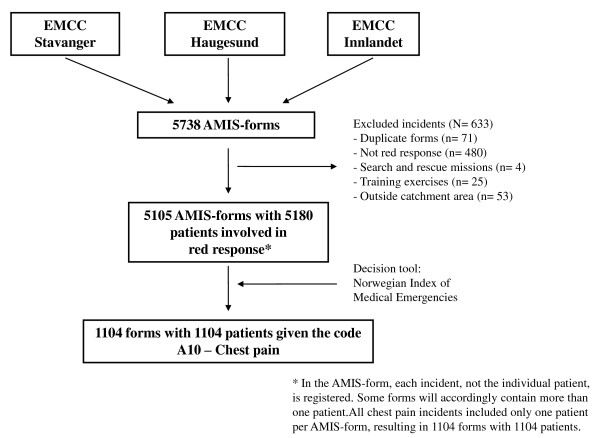
**Flow chart of AMIS forms received for registration, with both excluded and included incidents**.

A total of 5 738 AMIS-forms were collected from the three participating EMCC-districts during the three month period, of which 5 105 AMIS-forms with 5 180 patients (each form could include more than one patient) were included in the study (Figure 1). 1 104 of the patients (21%) were assigned the code A10 - Chest pain according to the Index, corresponding to a rate of 5.4 (95% CI 5.3-5.6) chest pain cases reported to the EMCCs per 1000 inhabitants per year. Further analyses are based on the 1 104 patients with code A10 - Chest pain.

**Figure 2:**
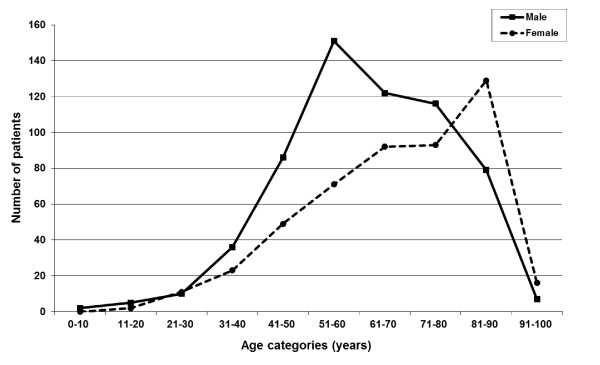
**Study patients with acute chest pain, by age and gender**.

The patients' age ranged from 4 to 97 years (median (25^th^-75^th ^percentile): 65 (53-79)), 56% males with a median age of 61 (25^th^-75^th ^percentile: 52-75), and 44% females with median age 70 (25^th^-75^th ^percentile: 56-82). The males were significantly younger than the females (p < 0.0001), and males dominated the age group 30-69 years with 63%, while the females constituted the majority (54%) in the age group > 70 years (Figure 2). There were only minor differences in the distribution of patients around-the-clock.

The primary care doctor on-call was alerted by radio alarm in 351 (36%) of the cases, of which the doctor responded with an emergency call out in about a third. The doctors' responses and choices of action are shown in Table 1. In 417 (38%) of the medical emergencies with chest pain as the main symptom, the caller to the EMCC was a next-of-kin, in 173 (16%) the patient, and a layperson made the call in 61 (6%). A physician called directly to the EMCC for assistance in 108 (11%) of the cases, while the call came from other health personnel in 314 (29%) of the cases.

**Table 1:** Alerting of doctors with their response, prehospital response time, air ambulance involvement and to where the patients were brought by NACA-score

						NACA Scores				
	
	Total		0-1		2-3		4-6		7	
	**N**	**(%)**	**N**	**(%)**	**N**	**(%)**	**N**	**(%)**	**N**	**(%)**
*Doctor was the caller*	108	(11)	4	(5)	65	(10)	39	(16)	0	(0)
*Doctors alerted*	351	(36)	36	(41)	214	(34)	95	(39)	6	(60)
*Doctors neither caller or alerted *	512	(53)	47	(54)	352	(56)	109	(45)	4	(40)
Total	971	(100)	87	(100)	631	(100)	243	(100)	10	(100)
										
*Doctors' response when alerted*										
Call out	109	(33)	7	(21)	57	(29)	39	(43)	6	(100
Awaiting further notice	138	(42)	16	(47)	90	(46)	32	(36)	0	(0)
Occupied with other patient(s)	2	(1)	0	(0)	2	(1)	0	(0)	0	(0)
No contact/response from doctor	9	(3)	1	(3)	3	(1)	5	(6)	0	(0)
Consultation with hospital	69	(21)	10	(29)	45	(23)	14	15)	0	(0)
Total	327	(100)	34	(100)	197	(100)	90	(100)	6	(100)
										
*Prehospital response time*										
0-9 minutes	276	(30)	20	(23)	176	(29)	76	(33)	4	(57)
10-19 minutes	413	(45)	38	(44)	287	(47)	86	(38)	2	(29)
> 20 minutes	237	(25)	28	(33)	143	(24)	65	(29)	1	(14)
Total	926	(100)	86	(100)	606	(100)	227	(100)	7	(100)
										
*Air ambulance requested*										
Yes	56	(6)	0	0)	13	(2)	39	(16)	4	(40)
No	915	(94)	87	(100)	618	(98)	204	(84)	6	(60)
Total	971	(100)	87	(100)	631	(100)	243	(100)	10	(100)
										
*Air ambulance response*										
Helicopter with anaesthetist sent	34	(69)	0	(0)	5	(45)	25	(74)	4	(100)
Ground vehicle with anaesthetist sent	9	(18)	0	(0)	5	(45)	4	(12)	0	(0)
Awaiting further notice	1	(2)	0	(0)	1	(9)	0	(0)	0	(0)
No flight due to weather condition	4	(8)	0	(0)	0	(0)	4	(12)	0	(0)
No flight due to technical problem	1	(2)	0	(0)	0	(0)	1	(3)	0	(0)
Total	49	(100)	0	(0)	11	(100)	34	(100)	4	(100)
										
*Patients brought to*										
Casualty clinic	143	(15)	46	(53)	95	(15)	2	(1)	0	(0)
Hospital via casualty clinic	121	(13)	0	(0)	108	(17)	13	(5)	0	(0)
Directly hospital, doctor involved	373	(39)	0	(0)	216	(34)	157	(65)	0	(0)
Directly hospital, doctor not involved	230	(24)	0	(0)	161	(26)	69	(29)	0	(0)
Patient remained on site	87	(9)	38	(44)	49	(8)	0	(0)	0	(0)
Deceased	10	(1)	0	(0)	0	(0)	0	(0)	10	(100)
Taken care of by other	2	(~0)	2	(2)	0	(0)	0	(0)	0	(0)
Total	966	(100)	86	(100)	629	(100)	241	(100)	10	(100)

**Figure 3:**
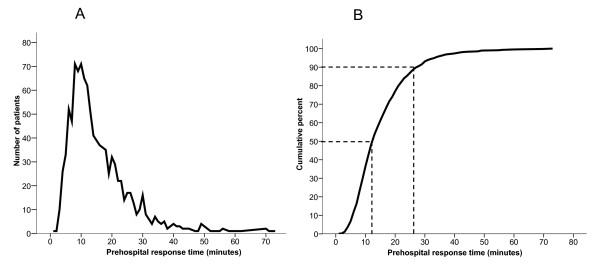
**Prehospital response time, defined as the time period from the caller calls the emergency number 113 until the nearest available ambulance resource reaches the patient**. a. Number of patients reached per minute b. Number of patients reached, cumulative percentage. Presented with 50- and 90-percentiles.

Median prehospital response time was 13 minutes (95% CI 9-20), and over 90% of the patients were reached by an ambulance in less than 30 minutes. Figure 3 shows the number of patients reached per minute (Figure 3a) and cumulative by percentage (Figure 3b).

**Figure 4:**
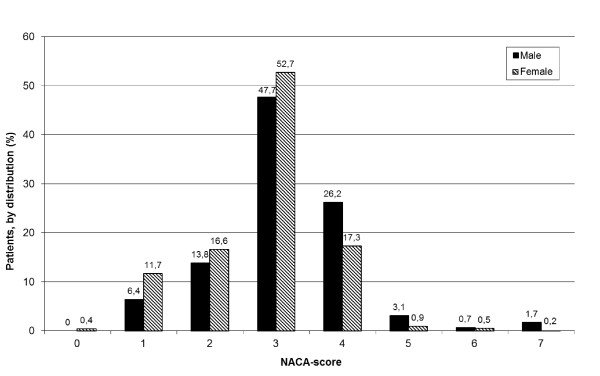
**Severity of illness (NACA-scores) in study patients (distribution) with acute chest pain, by gender**.

NACA-score could be classified in 971 (88%) of the patients (table 1), with 87 (9%) given NACA-score 0 or 1, indicating no illness or an illness not requiring medical attention. Overall, the female patients were given lower NACA-scores than the male patients, indicating less severe symptoms (p < 0.001), and in the group NACA 1, females constituted 59% of the patients (p < 0.01). Males dominated among the patients given NACA 4-6 (67% of the 163 patients, p < 0.001). Among the 10 patients who were dead, nine were male (p < 0.05). Figure 4 shows severity of illness (NACA-scores) in study patients, by gender.

Table 1 also describes the patients' severity of illness, represented by NACA-score stratified by whether the doctor was alerted by radio, doctor's response to the alarm, prehospital response time and involvement of air ambulance services. Severity of illness did not seem to affect whether or not the doctor was alerted by radio alarm, but the doctors' call out rate generally increased with the patients' severity of illness, with a call out in one of five patients with NACA 0-1, compared to 43% of the patients with NACA 4-6. Increasing NACA-score showed a tendency towards shorter prehospital response time, but the association between increasing NACA-score and shorter prehospital response time was not significant (p = 0,07).

Air ambulance was alerted in 56 (6%) of the cases, and a helicopter with an anaesthetist was sent to assist in 34 (3%) of the patients. Air ambulance service was not requested in any patients with NACA 0-1. In the group with potentially or definitely critically ill patients (NACA 4-6), a helicopter was requested in 16% of the cases, and actually sent to assist in 10%.

Analyses of the patients' whereabouts revealed that the large majority of the patients with acute chest pain categorised as "red response" were residing at home or at private facilities, 9% were in public areas and 6% at their general practitioner's surgery when the red response was triggered (table 1). The vast majority of the patients were admitted to a hospital for further investigation and/or treatment (N = 825, 76%), either via the casualty clinic (12%) or directly with (39%) or without (25%) being examined by a doctor. Of the 267 patients who were not admitted, 155 (58%) received final treatment at the casualty clinic, while 100 (37%) patients were not brought to a doctor for further investigation or treatment.

The cases were also classified with an ICPC-2 code, with the codes A11 "Chest pain" (56%) and K01 "Heart pain" (32%) constituting the vast majority. The remainder 12% were spread over 35 different ICPC-2 codes, with A06 "Fainting/syncope" accounting for 3% of the cases, and R02/R04 "Dyspnoea/Breathing problem" 2%. An ICPC-2 code from the psychiatry-chapter (P01-P29) was used in 1%.

## Discussion

### Summary of main findings

This prospective population based study showed an estimated rate of 5.4 acute chest pain cases involved in a red response per 1000 inhabitants per year. This corresponds to approximately 10 patients with acute chest pain in need of immediate medical help each week in an out-of-hours district covering 100.000 inhabitants. Over 20% of all contacts to the EMCCs ending in a red response involved chest pain as the main symptom. Males constituted a majority of the patients, and were significantly younger than the females. NACA-scores indicated that only a fourth of the patients were in a potentially or definitely life-threatening medical situation (NACA ≥ 4), but more than three quarters were admitted to a hospital for further investigation and treatment.

### Strengths and weaknesses of the study

The main strength of our study is the large register of data collected, where we were able to prospectively collect a complete material of more than 5 000 red responses during the three month period, based on a population close to 820 000 inhabitants, about 20% of the Norwegian population. Limitations include NACA-scores in most of the cases being assessed retrospectively based on medical records, which might give a lower accuracy when registering the severity of the illness. Severity assessment in patients with chest pain can be difficult from medical records alone, but the records included the patients' symptoms and clinical findings, making it possible to achieve reliable registrations. Ideally the study would have included on-going clinical evaluation by the physicians on-site, in addition to results and diagnoses from the investigations for the patients admitted to the hospital. Our results are based solely on patients in an emergency situation defined by the EMCCs using the Index (red response), and thus undertriaged patients would not be included. Patients with chest pain assigned with a yellow response might be at risk of being undertriaged ("false negatives"), supporting the need for further studies on all patients with chest pain outside hospitals. The degree of urgency was set by trained nurses using the Norwegian Medical Index of Emergencies, but little is known about the validity of the Index and how the Index is used in the different EMCCs. A throughout evaluation and validation of the Index is needed.

### Previous studies

The rate of acutely ill patients with chest pain in our study is similar to the findings in two other studies from Norway, reporting rates of 4.8 [7] and 5.4 [8]. The difference in median age between the genders, with the males being significantly younger, is in accordance with previous studies [14]. Recent studies from the UK [2,3] and the US [15] have shown that around 10% of calls to emergency medical dispatch systems involve acute chest pain. A Norwegian publication from 2009 [16] showed that 22% of all the calls to the emergency number 113 ended in a red response, and it is intended that most of the chest pain incidents will be classified as a red response. In our study this would indicate that approximately 5% of all calls to the EMCCs involved chest pain as the main complaint, given that all incidents with chest pain were classified as a red response.

### Meaning of study

A substantial number of the patients were not in a life threatening medical situation. This sheds light on the challenges for the EMCCs in deciding the appropriate level of response in patients with acute chest pain. Overtriage is to some extent both expected and desirable to intercept all patients in need of immediate help, but it is also well known that overtriage is resource demanding. Almost 10% of the patients were not brought to a doctor for further investigation or treatment. This indicates that the patient's medical condition was not as severe as initially assessed, supported by our results showing that all of these patients were given a NACA-score of ≤ 3. Norwegian health authorities and cardiologists have called attention to the importance of patients calling the three digits emergency number "113" directly when experiencing acute chest pain. Our study shows that in almost half of the calls to EMCC the call was made from health personnel, representing a possible system delay for patients with chest pain of cardiac origin in need of immediate diagnosis and treatment. Still, as the vast majority of patients with acute chest pain seem not to be in need of immediate hospital care, the primary care doctor on-call at the casualty clinic should still play an important role after the first contact to the EMCC. Primary care doctors are usually experienced in differentiating between severe and non-severe illness. As a group, they also hold a clinical background and competence making them a valuable asset in the initial management of patients with acute chest pain outside hospitals.

A white paper concerning the organisation of the emergency services in Norway [17] have defined recommended minimum requirements for prehospital response times in red response missions. An ambulance should have reached 90% of the patients within 8 minutes in urban districts, and 25 minutes in rural districts. Our results show that 87% of all patients with acute chest pain are reached within 25 minutes, but only 23% within 8 minutes. This might partly be explained by the fact that a considerable number of patients from the study population live in rural districts. But it also sheds light on the reality in Norwegian prehospital emergency medicine, which shows that we are still quite far from meeting the political aims concerning minimum requirements for prehospital response time [18].

## Conclusions

The majority of patients with acute chest pain were admitted to a hospital for further investigation, but only a quarter of the patients were assessed prehospitally to have a severe illness. Little is still known about the extent of patients with chest pain as their main symptom outside hospitals in Norway, including diagnostic measures, how they are treated and rates of admission to the hospital.

## Competing interests

The authors declare that they have no competing interests.

## Authors' contributions

EZ and SH planned and established the project, including the procedures for data collection. RAB designed the paper, performed the analyses and drafted the first manuscript. All authors took part in rewriting and approved the final manuscript.

## Funding

The project was partly funded by the National Centre for Emergency Primary Health Care, Uni Health, Bergen. RAB has received a research grant from the Norwegian Medical Association's fund for Research in General Practice.

## Pre-publication history

The pre-publication history for this paper can be accessed here:

http://www.biomedcentral.com/1471-227X/11/9/prepub

## References

[B1] Zakariassen E Burman RA Hunskaar S The epidemiology of medical emergency contacts outside hospitals in Norway-a prospective population based study Scand J Trauma Resusc Emerg Med 18 9 10.1186/1757-7241-18-9 PMC2836273 20167060

[B2] Deakin CD Sherwood DM Smith A Does telephone triage of emergency (999) calls using Advanced Medical Priority Dispatch (AMPDS) with Department of Health (DH) call prioritisation effectively identify patients with an acute coronary syndrome? An audit of 42,657 emergency calls to Hampshire Ambulance Service NHS Trust Emerg Med J 2006 23 3 232 5 10.1136/emj.2004.022962 16498168 PMC2464449

[B3] Clawson J Olola C Heward A The Medical Priority Dispatch System's ability to predict cardiac arrest outcomes and high acuity pre-hospital alerts in chest pain patients presenting to 9-9-9 Resuscitation 2008 78 3 298 306 10.1016/j.resuscitation.2008.03.229 18562077

[B4] Cayley WE Jr Diagnosing the cause of chest pain Am Fam Physician 2005 72 10 2012 21 16342831

[B5] Buntinx F Knockaert D Bruyninckx R Chest pain in general practice or in the hospital emergency department: is it the same? Fam Pract 2001 18 6 586 9 10.1093/fampra/18.6.586 11739341

[B6] Norwegian Medical Association Norsk indeks for medisinsk nødhjelp 2005 (Norwegian Index of Emergency Medical Assistance) Stavanger: The Laerdal Foundation for Acute Medicine, 2.1

[B7] Rortveit S Hunskar S Medical emergencies in a rural community Tidsskr Nor Laegeforen 2009 129 8 738 42 19373298 10.4045/tidsskr.08.0019

[B8] Aune E Steen-Hansen JE Hjelmesaeth J Prehospital diagnosis and treatment of acute myocardial infarction in Vestfold Tidsskr Nor Laegeforen 2004 124 23 3058 60 15586187

[B9] Folkestad EH Gilbert M Steen-Hansen JE Urgent calls-prehospital response time in Vestfold and Troms in 2001 Tidsskr Nor Laegeforen 2004 124 3 324 8 14963502

[B10] Yang JJ Dreyer K Eielsen O Definisjonskatalog for AMK/LV-sentraler 1999 1. utg. KITH Rapport 3/99. Trondheim: Kompetansesenteret for IT i helsesektoren

[B11] Culley LL Henwood DK Clark JJ Increasing the efficiency of emergency medical services by using criteria based dispatch Ann Emerg Med 1994 24 5 867 72 10.1016/S0196-0644(54)00223-5 7978559

[B12] The National Committee on Aeronautics (NACA) http://www.medal.org/visitor/www/Active/ch29/ch29.01/ch29.01.13.aspx

[B13] World Health Organization: International Classification of Primary Care, (ICPC-2) http://www.who.int/classifications/icd/adaptations/icpc2/en/index.html

[B14] Bruyninckx R Van den Bruel A Aertgeerts B Why does the general practitioner refer patients with chest pain not-urgently to the specialist or urgently to the emergency department? Influence of the certainty of the initial diagnosis Acta Cardiol Acta Cardiol 2009 64 2 259 265 10.2143/AC.64.2.2036147 19476121

[B15] Sporer KA Youngblood GM Rodriguez RM The ability of emergency medical dispatch codes of medical complaints to predict ALS prehospital interventions Prehosp Emerg Care 2007 11 2 192 8 10.1080/10903120701205984 17454806

[B16] National Centre on Emergency Communication in Health 2009 Kartlegging av virksomhetsdata fra nødmeldesentraler i helse. Driftsdata fra oppdragshåndtering. Kokom

[B17] Ministry of Health and Care Services: Stortingsmelding 43 (1999-2000) Om akuttmedisinsk beredskap. (About emergency preparedness) http://www.regjeringen.no/nb/dep/hod/dok/regpubl/stmeld/19992000/stmeld-nr-43-1999-2000-.html?id = 193493

[B18] Office of the Auditor General of Norway: Riksrevisjonens undersøkelse av akuttmedisinsk beredskap i spesialisthelsetjenesten. (The OAG's investigation of emergency medical preparedness in the specialist health service. English summary) http://www.riksrevisjonen.no/en/Reports/Pages/Dokumentbase_Eng_Doc_3_9_2005_2006.aspx

